# ﻿Resolving the taxonomic enigma of *Nesocaryum stylosum* (Boraginaceae): phylogenetic evidence for its reclassification as *Cryptantha stylosa*

**DOI:** 10.3897/phytokeys.269.176263

**Published:** 2026-01-13

**Authors:** Michael G. Simpson, Makenzie E. Mabry, Paul D. Blischak, Tim Böhnert, C. Matt Guilliams, Kristen E. Hasenstab-Lehman, Federico Luebert, Nicolás García, Álvaro Castañeda, Maximilian Weigend, Rosa A. Scherson

**Affiliations:** 1 Department of Biology, San Diego State University, San Diego, California 92182, USA San Diego State University San Diego United States of America; 2 Florida Museum of Natural History, University of Florida, Gainesville, Florida 32611, USA University of Florida Gainesville United States of America; 3 Bayer Crop Science, Chesterfield, Missouri, 63017 Bayer Crop Science Chesterfield United States of America; 4 Bonn Institute of Organismic Biology, University of Bonn, Meckenheimer Allee 170, 53115 Bonn, Germany Universität Bonn Bonn Germany; 5 Department of Conservation and Research, Santa Barbara Botanic Garden, Santa Barbara, California 93105, USA Santa Barbara Botanic Garden Santa Barbara United States of America; 6 Laboratorio de Evolución y Sistemática & Herbario EIF, Departamento de Silvicultura y Conservación de la Naturaleza, Universidad de Chile, Santiago, Chile Universidad de Chile Santiago Chile; 7 Departamento de Ciencias Ambientales y Recursos Naturales Renovables, Universidad de Chile, Santiago, Chile University of Bonn Bonn Germany

**Keywords:** Chile, genome skim, Isla San Ambrosio, *Maritimae* clade, molecular phylogenetics, series *Maritimae*, series *Muricatae*, taxonomy

## Abstract

The classification of *Nesocaryum stylosum* (Boraginaceae) has remained unresolved for nearly a century. This species is endemic to Isla San Ambrosio, a small island located approximately 900 km due west of the coast of central Chile. Ivan M. Johnston transferred the species from the genus *Heliotropium* to the monotypic *Nesocaryum* in 1927 but noted that, despite its quite different and rather unique vegetative, inflorescence, and calyx morphology, its unit fruits (nutlets/eremocarps) resemble those of *Cryptantha*. Here, we review the morphology and taxonomic history of *N. stylosum* and provide DNA sequence data that support its placement in *Cryptantha*, for which we propose the new combination *Cryptantha stylosa*. Our data also support the placement of this species within the *Maritimae* clade, a monophyletic group of *Cryptantha* species that is phylogenetically distinct from the bulk of the genus. We propose an expanded membership of the *Maritimae* clade comprising up to 19 species: eight species (13 minimum-rank taxa) from North America and 12 species (12 minimum-rank taxa) from South America, including *Cryptantha stylosa*, with one taxon occurring on both continents. We further review evidence bearing on the biogeographic and evolutionary history of *Cryptantha stylosa* and its putative closest relatives and identify the need for additional research within the group.

## Abstract (es)

La clasificación de *Nesocaryum stylosum* (Boraginaceae) ha permanecido sin resolverse durante casi un siglo. Esta especie es endémica la isla de San Ambrosio, una pequeña isla ubicada aproximadamente a 900 km al oeste de la costa de Chile central. Ivan M. Johnston transfirió la especie desde *Heliotropium* al monotípico *Nesocaryum* en 1927, pero observó que, a pesar que su morfología vegetativa, de inflorescencia y de cáliz es bastante diferente y única, sus frutos unitarios (clusas/eremocarpos) se asemejan a los de *Cryptantha*. Aquí revisamos la morfología y la historia taxonómica de *N. stylosum* y proporcionamos datos de secuencia de ADN que respaldan su ubicación en *Cryptantha*, para la cual proponemos la nueva combinación *Cryptantha stylosa*. Nuestros datos también respaldan la ubicación de esta especie dentro del clado *Maritimae*, un grupo monofilético de especies de *Cryptantha* que es filogenéticamente distinto de la mayor parte del género. Proponemos una ampliación de la composición del clado *Maritimae*, con hasta 19 especies: ocho especies (13 taxones de rango mínimo) de Norteamérica y 12 especies (12 taxones de rango mínimo) de Sudamérica, incluyendo *Cryptantha stylosa*, con un taxón presente en ambos continentes. Además, revisamos la evidencia de la historia biogeográfica y evolutiva de *Cryptantha stylosa* y sus posibles parientes más cercanos, e identificamos la necesidad de investigación adicional dentro del grupo.

## ﻿Introduction

The genus *Cryptantha* Lehm. ex G.Don (Boraginaceae, subtribe Amsinckiinae, after Chacón et al. 2016), as currently delimited, consists of approximately 111 species and 126 minimum-rank taxa (the latter including the total number of accepted taxa, including varieties and subspecies, after Baldwin 2000), with 65 species native to North America, 47 species native to South America, and one of these species found on both continents (see Hasenstab-Lehman and Simpson 2012, Simpson et al. 2017a, Mabry and Simpson 2018, Amsinckiinae Working Group 2025). Even though the numerous species in the genus may be distinguished from one another by life history (perennial versus annual), stem habit and branching pattern and indument, inflorescence cymule number, leaf morphology, bract presence or absence and morphology, corolla limb width and fornix presence, absence, or color, and fruiting calyx shape, size, and indument, it is the morphology of the unit fruits, termed nutlets (also termed “eremocarps”; see Hilger 2014; Jeiter et al. 2018), that is often most important in delimiting taxa. The nutlets of *Cryptantha* species vary in number per fruit (1–4), heteromorphism (differences between nutlets within a fruit), size, shape, and sculpturing features, the latter including smooth, papillate, muricate or tuberculate, spinulose, and winged (see Simpson and Hasenstab 2009).

**Figure 1.**
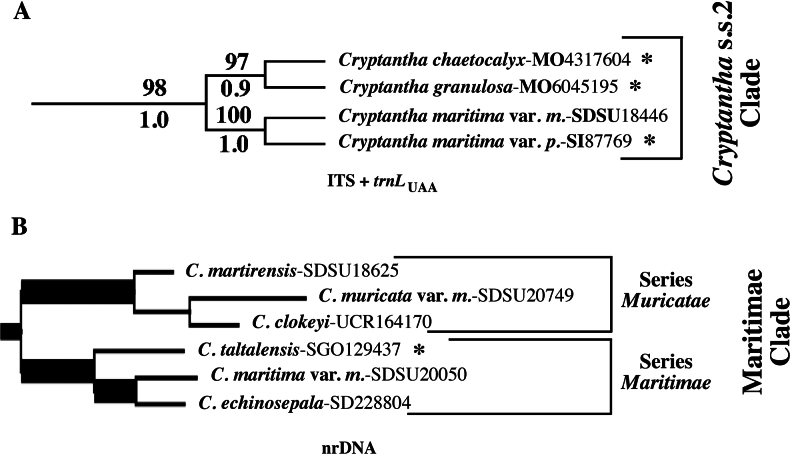
Cladograms, redrawn from original publications. **A.***Cryptantha* s.s.2 clade from Hasenstab-Lehman and Simpson (2012), based on ITS and cpDNA data and both analyses; **B.***Maritimae* clade from Simpson et al. (2017a), cistron analysis; chloroplast and mitochondrial analyses from that study are identical in topology. Series *Muricatae* and *Maritimae* are designated as in Table 1; see Discussion.

*Cryptantha*, as it has traditionally been circumscribed, has nutlets that bear a slit-like “ventral groove” positioned above and contiguous with the attachment scar. The molecular phylogenetic analyses of Hasenstab-Lehman and Simpson (2012), Simpson et al. (2017a), and Mabry and Simpson (2018) clarified generic circumscriptions within subtribe Amsinckiinae, to which *Cryptantha* belongs (see Chacón et al. 2016). These studies provided evidence for the segregation (and resurrection) of four genera that also have a ventral-groove attachment scar – *Eremocarya* Greene, *Greeneocharis* Gürke & Harms, *Johnstonella* Brand, and *Oreocarya* Greene – from the traditional circumscription of *Cryptantha* s.l. (note that a ventral groove evolved independently in some species of the genus *Amsinckia*). However, one additional clade of *Cryptantha* s.l. is phylogenetically separate from the bulk of *Cryptantha* taxa and from the four segregate genera mentioned above, a clade first reported by Hasenstab-Lehman and Simpson (2012). In that study, which used one chloroplast region and the nuclear cistron ITS genetic marker, the authors described what they termed the *Cryptantha* s.s.2 clade (Fig. 1A). The phylogenetic position of the *Cryptantha* s.s.2 clade varied depending on the type of analysis, ranging from being sister to *Cryptantha* s.s.1 (consisting of all other examined species in the genus) to being well separated and sister to a larger clade containing four other genera.

The phylogenetic study of subtribe Amsinckiinae by Simpson et al. (2017a) utilized sequence data from chloroplast DNA (cpDNA), mitochondrial DNA (mtDNA), and nuclear ribosomal DNA (nrDNA). This study also recovered a rogue group of *Cryptantha* species, which the authors informally termed the “*Maritimae* clade” (after *Cryptantha maritima* (Greene) Greene, a member of this clade). The authors interpreted this clade as equivalent to the *Cryptantha* s.s.2 clade of Hasenstab-Lehman and Simpson (2012) because of the shared membership of C. maritima var. maritima in both, but with expanded membership (Fig. 1B). However, in the Simpson et al. (2017a) analyses, three species corresponding to Johnston’s (1925) series *Muricatae* form a clade that is sister to a clade corresponding in part to Johnston’s series *Maritimae*. As in the earlier analysis, the *Maritimae* clade of Simpson et al. (2017a) also varied in phylogenetic position relative to other clades in subtribe Amsinckiinae depending on which of the three data types were analyzed; it is sister to *Eremocarya* with weak to mixed support in the mitochondrial and chloroplast DNA analyses, but sister to the remainder of the genus, i.e., *Cryptantha* s.s., with mixed support in the nuclear ribosomal (ITS) analysis.

**Figure 2.**
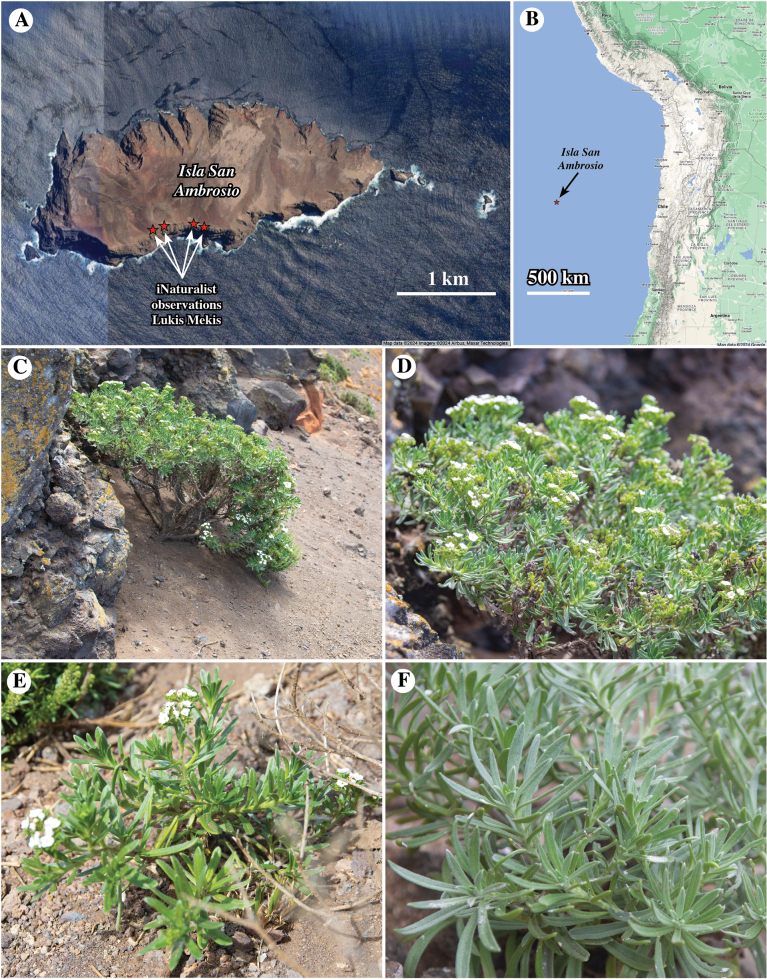
**A, B.** Map of Isla San Ambrosio, Chile, showing observations of *Nesocaryum stylosum*. **A.** Close-up satellite image showing the island and four localities of iNaturalist observations by Lukas Mekis (see Appendix 5); map from Google 2024, imagery 2024 Airbus, Maxar Technologies; **B.** Map showing the position of the island relative to South America, ca. 900 km west of the coast of Chile; map data Google 2024; **C–F.** Posted images of iNaturalist observations by Lukas Mekis, lukasmekis, some rights reserved (CC BY-NC); see Appendix 5 for full data: **C.** Whole plant, a small shrub (17 December 2021); **D.** Close-up of C (17 December 2021); **E.** Whole plant showing corymbose clusters of white flowers (17 December 2021); **F.** Close-up of vegetative shoots showing narrowly elliptic, gray-green, sericeous leaves (31 March 2022).

**Figure 3.**
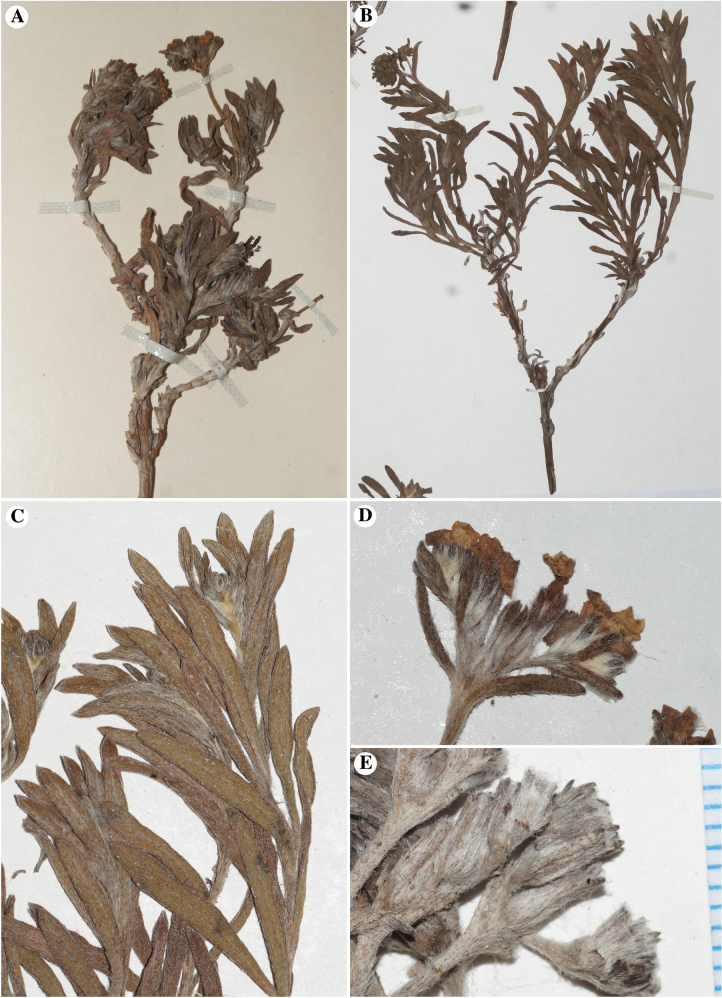
*Nesocaryum stylosum* herbarium sheet images. **A.** Holotype, E. [Enrique] Simpson s.n., 1869 (barcode SGO000004198 = accession SGO054406), showing a single whole plant; note woody stem; **B–E.** Specimen, N. Bahamande N. s.n., 21 August 1960 (accession SGO122886): **B.** One of several whole plants; **C.** Close-up of linear leaves; **D.** Inflorescence composed of two cymules arranged in a corymbose secondary unit; **E.** Developing fruits; note whitish, sericeous vestiture. All images were taken by Michael G. Simpson at the SGO herbarium, by permission.

**Figure 4.**
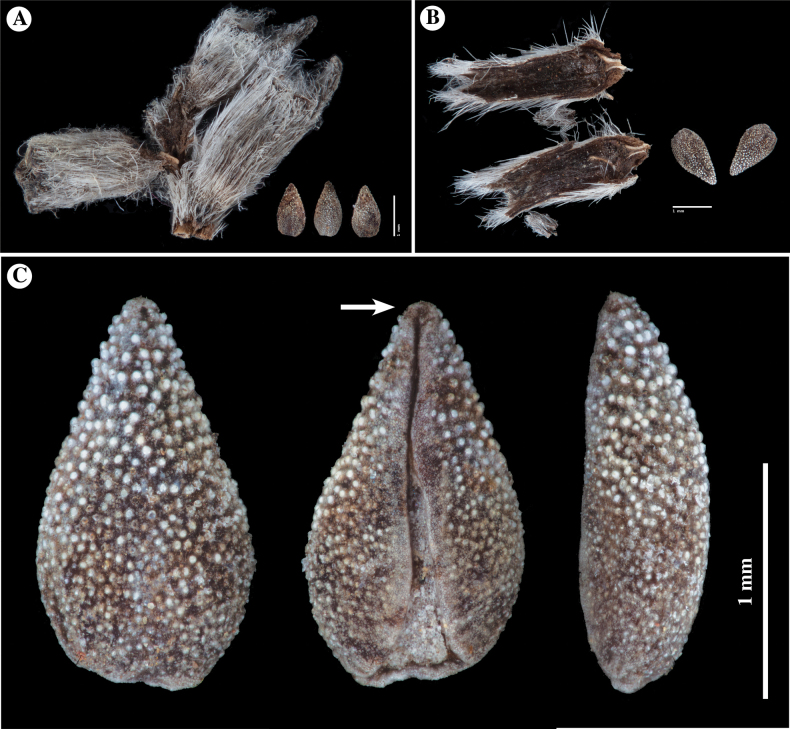
*Nesocaryum stylosum*, material from specimen N. Bahamande N. s.n., 21 August 1960 (accession SGO122886). **A.** Close-up of fruiting calyces and three nutlets from one fruit; **B.** Fruiting calyx opened, showing two nutlets within; **C.** Nutlet close-up, shown (left to right) in dorsal, ventral, and lateral views; note smaller papillae and larger, rounded, whitish tubercles. Arrow indicates slightly developed expansion of the apical tip of the ventral groove. All images were taken by Lee M. Simpson, San Diego State University; material removed from specimens at the SGO herbarium, by permission.

Another member of Boraginaceae found in South America, the monotypic *Nesocaryum stylosum* (Phil.) I.M.Johnst., has puzzled taxonomists for nearly a century. This species is endemic to Isla San Ambrosio, a small (ca. 3.7 km^2^), rugged, uninhabited island located 900 km due west of the coast of central Chile (Fig. 2A, B). This plant species was originally described by Philippi (1870) as *Heliotropium stylosum* Phil. (see Appendix 1 for the protologue description). The species resembles many *Heliotropium* species in being a shrub with clustered (corymbose) white flowers (Figs 2C–F, 3A, B, D). Leaf surfaces and calyces are covered with whitish, densely strigose indument (Figs 2F, 3C, E, 4A). The epithet *stylosum* undoubtedly refers to the unique (“singularem,” in Philippi’s description) elongate style, described as twice as long as the ovary in the protologue (Appendix 1).

In 1927, Johnston transferred this species of *Heliotropium* to its own genus, which he named *Nesocaryum* I.M.Johnst. (after neso-, island + caryum, fruit) (see Appendix 2 for the protologue description). Johnston (1927: 75) stated that *N. stylosum* was “evidently derived from *Cryptantha*,” given that the fruit (Fig. 4C) was “indistinguishable” from members of that genus. However, he placed the species in a monotypic genus because of its unique, even within Boraginaceae, calyx (Fig. 4A, B) and corolla morphology. The calyx is described as cylindrical with an elongate, abaxial adnate bract, and the corolla tube has “5 pairs of knife-like lamellae” at the base. Johnston considered *Nesocaryum stylosum* to be a derivative of a member of Cryptantha sect. Krynitzkia ser. Barbigerae (see Discussion).

In light of the long-standing uncertainty surrounding the affinities of *Nesocaryum stylosum*, the primary objective of this study is to analyze sequence data to evaluate its phylogenetic position and biogeographic history. We first test whether the species does indeed nest within the genus *Cryptantha*, as implied by nutlet morphology, or within another group of subtribe Amsinckiinae of Boraginaceae. If nested within *Cryptantha*, we then ask which taxa represent its closest relatives. Is *Nesocaryum stylosum* closely related to members of sect. Krynitzkia ser. Barbigerae, as suggested by Johnston (1927)? An overarching goal of this study is to evaluate the relationship of *Nesocaryum stylosum* to other members of this complex of plants and to address strongly supported results by updating the taxonomy of this species.

## ﻿Methods

### ﻿Sample collection and DNA extraction

Obtaining suitable material for DNA extraction from *Nesocaryum* proved challenging. An attempt to extract DNA from a specimen collected in 1960 was unsuccessful. However, using more recently collected material (*Christian López Chamorro* s.n., September 2018; EIF17397), we successfully extracted and sequenced DNA. Including *Nesocaryum*, we sequenced 94 new samples in this study across the Amsinckiinae. These new samples included several taxa not previously sequenced in Simpson et al. (2017a), including new members of the *Maritimae* clade. We relied on material available to us from herbarium specimens and silica-dried leaves from live material. To further expand these data, we downloaded sequence data for 142 samples from the Sequence Read Archive (SRA), representing data from Simpson et al. (2017a). These combined datasets were used for phylogenetic analysis, building upon the sequence data of Simpson et al. (2017a). Documentation of the new samples used for sequencing is provided in Appendix 3.

For DNA extraction, dried silica material was ground into a fine powder using a multi-sample Bead Beater tissue homogenizer and extracted using a modified cetyltrimethylammonium bromide (CTAB) protocol with the following modification: incubation in CTAB extraction buffer with proteinase K at 65 °C for 3–4 h, followed by overnight precipitations. Half of the samples underwent a final cleaning step using a Zymo DNA Clean and Concentrator-25 kit (Zymo Research, Irvine, CA, USA). DNA extractions were quantified on a Qubit fluorometer using the Qubit Double-Stranded High Sensitivity Assay Kit (Invitrogen, Carlsbad, CA, USA) to assess genomic DNA quantity. DNA quality was evaluated by visualization on an agarose gel following electrophoresis.

### ﻿Library preparation, size selection, library quantification, and sequencing

A run of 94 samples was performed at the University of California at Riverside Genomics Core in a single lane on an Illumina NextSeq2000 platform, yielding an average of 1,275,636 reads per sample (range: 849–4,040,175). This produced single-end 100 bp reads with an average insert size of 250 bp. All reads are deposited in the Short Read Archive of the NCBI (https://www.ncbi.nlm.nih.gov; see Appendix 3). Using high-throughput genome skimming sequencing (see Ripma et al. 2014), we obtained chloroplast, cistron, and mitochondrial genome data.

Read recovery varied substantially among samples, likely due to differences in DNA input concentrations across libraries. In addition, many sequences were fragmentary, possibly reflecting the degraded nature of DNA from older herbarium specimens (Appendix 3).

### ﻿Data analysis

We combined the sequence results of this study with those of Simpson et al. (2017a), yielding an initial total of 236 samples. One specimen of *Cryptantha taltalensis* I.M.Johnst. (SGO129437), included in the combined analyses, had previously been identified as *C. subamplexicaulis* (Phil.) Reiche in Simpson et al. (2017a) and Mabry and Simpson (2018); see Appendix 3. This name change resulted from a subsequent study of original material from that specimen and annotation by Pablo Moroni and Michael Simpson.

Raw sequencing reads were processed using CAPTUS (v1.4.8; Ortiz et al. 2023; https://github.com/edgardomortiz/Captus.git) to perform data cleaning, de novo read assembly, and targeted locus extraction. Initial quality control and adapter trimming were conducted using *captus clean*. Cleaned reads were subsequently assembled de novo using *captus assemble*. Following assembly, target regions of interest were extracted from assembled contigs using *captus extract*. This step employed a paralog tolerance of 1.5 and included mitochondrial targets specified by the parameter SeedPlantsMIT, as well as a custom full-chloroplast target derived from *Echium plantagineum* L. (Boraginaceae; Carvalho Leonardo et al. 2023), divided into 21 fragments designed to avoid breaks across gene boundaries. We also created a custom target for the cistron using assembly statistics to identify long contigs potentially spanning the complete region. For the sample *Cryptantha decipiens* (M.E.Jones) A.Heller (voucher SDSU20014 of Simpson et al. 2017a), a custom Python script was used to extract the cistron region from the contig NODE_57793_length_8014_cov_228.7687_k_95_flag_1, spanning positions 672–6471. Because this region was located on the negative strand, the extracted sequence was reverse-complemented. The resulting loci were subsequently aligned using *captus align*, which generated alignments for each marker type. For all analyses, we used the trimmed, naïve alignments produced by CAPTUS to reduce ambiguous regions and improve alignment quality. The 20 fragments of the full chloroplast genome and the 29 mitochondrial gene alignments were concatenated. Interactive HTML reports summarizing quality metrics and statistics for each step of data processing – cleaning, assembly, locus extraction, and alignment – along with target and tree files, have been deposited in Zenodo (https://doi.org/10.5281/zenodo.17246597).

Phylogenetic analyses were performed using IQ-TREE (v2.2.2.7; Minh et al. 2020). Maximum likelihood (ML) trees were inferred separately for the cistron, the full chloroplast genome, and concatenated mitochondrial genes. For the cistron alignment, IQ-TREE was run with the command-line options -nt AUTO to determine the optimal number of threads, -B 1000 to generate 1,000 ultrafast bootstrap replicates for node support, and -m MFP to enable ModelFinder Plus for automatic model selection. Analyses of the full chloroplast genome and concatenated mitochondrial genes were conducted under partitioned models. For these datasets, IQ-TREE was run with the alignment file in Nexus format and an accompanying partition file specifying gene or region boundaries. Commands included -nt AUTO, -B 1000 for ultrafast bootstrap support, —alrt 1000 for 1,000 SH-like approximate likelihood ratio test replicates, and -m MFP+MERGE to allow model selection with automatic merging of partitions based on similarity. Samples were excluded from phylogenetic analyses if they had 10 or fewer loci in the mitochondrial gene dataset or fewer than 10 recovered regions in the full chloroplast genome assembly (see Appendix 3). All scripts and code used for data processing and phylogenetic analyses are available in a public GitHub repository (https://github.com/mmabry/Nesocaryum_Boraginaceae.git). All trees were rooted using sequence data from the outgroup *Myosotis laxa* Lehm. (SBBG132391; see Simpson et al. 2017a).

### ﻿Distribution mapping

To generate distribution maps, georeferenced data points for all members of the series *Maritimae* (see below) were downloaded from the Global Biodiversity Information Facility (GBIF.org 2024a–p). A total of 5,518 GBIF records were mapped and are listed by series and species in Appendix 4. These records were not taxonomically verified by the authors. In addition, iNaturalist (https://www.inaturalist.org) observations of *Nesocaryum stylosum* (Appendix 5) were mapped in detail. Maps were generated using R v4.3.1, with duplicate records removed and occurrence points plotted on a custom Google Map (Kahle and Wickham 2013).

## ﻿Results

**Figure 5.**
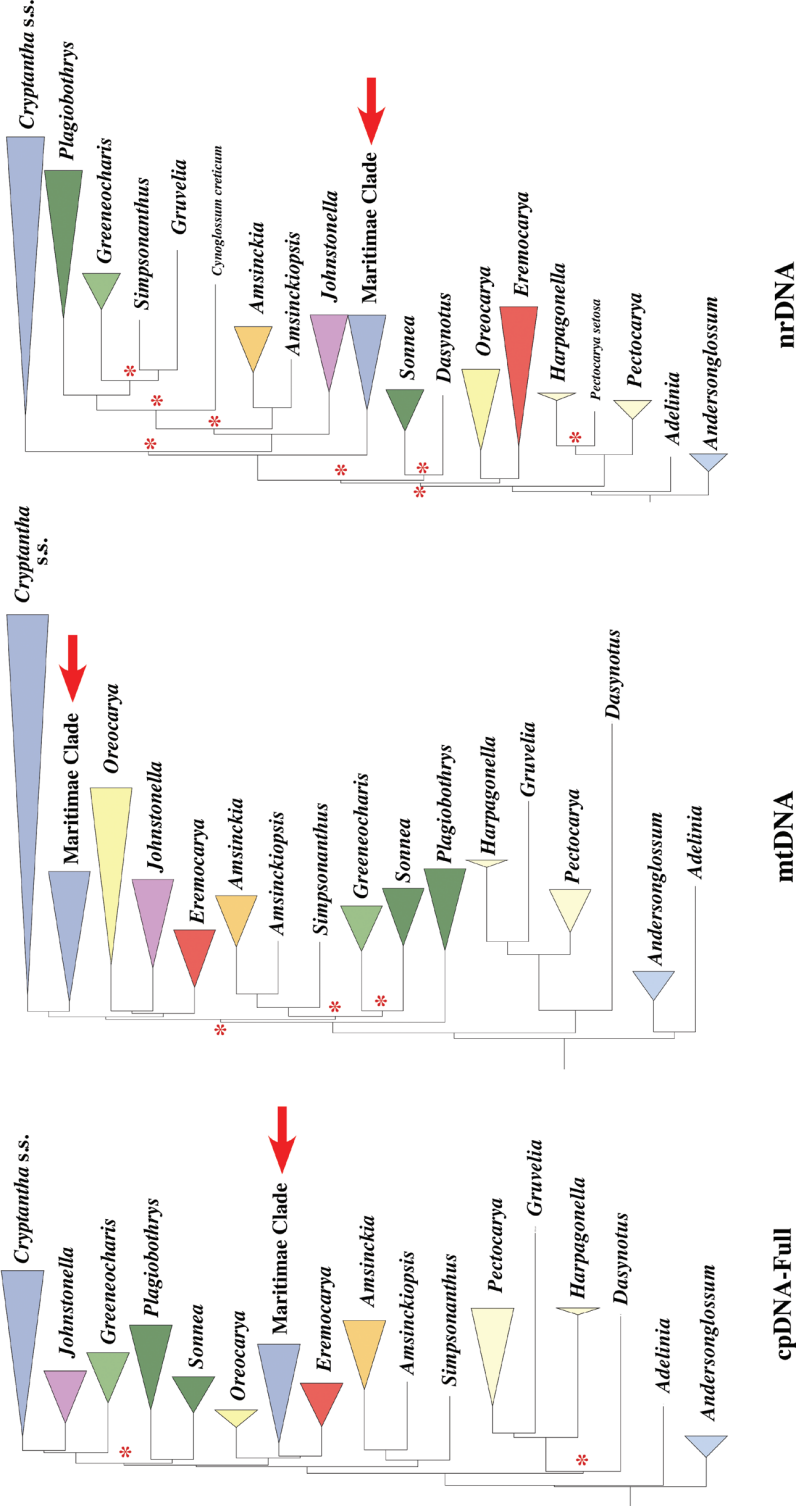
Cladograms of subtribe Amsinckiinae using the three DNA sequence datasets of this study: chloroplast (cpDNA), mitochondrial (mtDNA), and cistron (nrDNA). Genera and clades are represented by colored triangles, all robustly supported except for some taxa in the nrDNA analysis. Relatively short branches and weak support along the backbone of these analyses are indicated, with an asterisk representing nodal bootstrap support values <90%. Note differences in interrelationships among major clades and genera. The *Maritimae* clade (red arrows) is sister to *Cryptantha* s.s. only in the mtDNA analysis. All trees were rooted using sequence data from the outgroup *Myosotis laxa* Lehm. (SBBG132391).

The three phylogenetic analyses, using sequences from the full chloroplast (cpDNA), mitochondria (mtDNA), and cistron (nrDNA), recovered the same genera and clades identified by Simpson et al. (2017a), including the *Maritimae* clade, all of which were individually well supported, with 100% bootstrap support values (Fig. 5). The only exception was the nrDNA analysis, in which *Pectocarya* is not monophyletic (with *P. setosa* A.Gray more closely related to *Harpagonella* A.Gray), *Cynoglossum creticum* Mill. is nested within the Amsinckiinae (being outside the subtribe in the other two analyses), and not all genera and clades are well supported. These nrDNA results mirror those of Simpson et al. (2017a), with interrelationships among genera and clades varying depending on the sequence data used. Thus, the backbone of the Amsinckiinae is weak in terms of bootstrap support values (Fig. 5). The full cladograms for each of the three sequence datasets are shown in Suppl. materials 1–3.

This variability in genus and clade interrelationships is exemplified by the *Maritimae* clade, whose topological position shifts among the three analyses. The *Maritimae* clade is sister to *Cryptantha* s.s. in the mtDNA analysis but considerably more distant from *Cryptantha* s.s. in the cpDNA analysis, in which it is sister to *Eremocarya* with good support but a shallow branch, and in the nrDNA analysis, in which it is sister to a complex of genera with weak support (Fig. 5). Interestingly, in the analyses of Simpson et al. (2017a), the *Maritimae* clade is sister to *Cryptantha* s.s. only in the nrDNA analysis, not in the mtDNA analysis as observed here.

**Figure 6.**
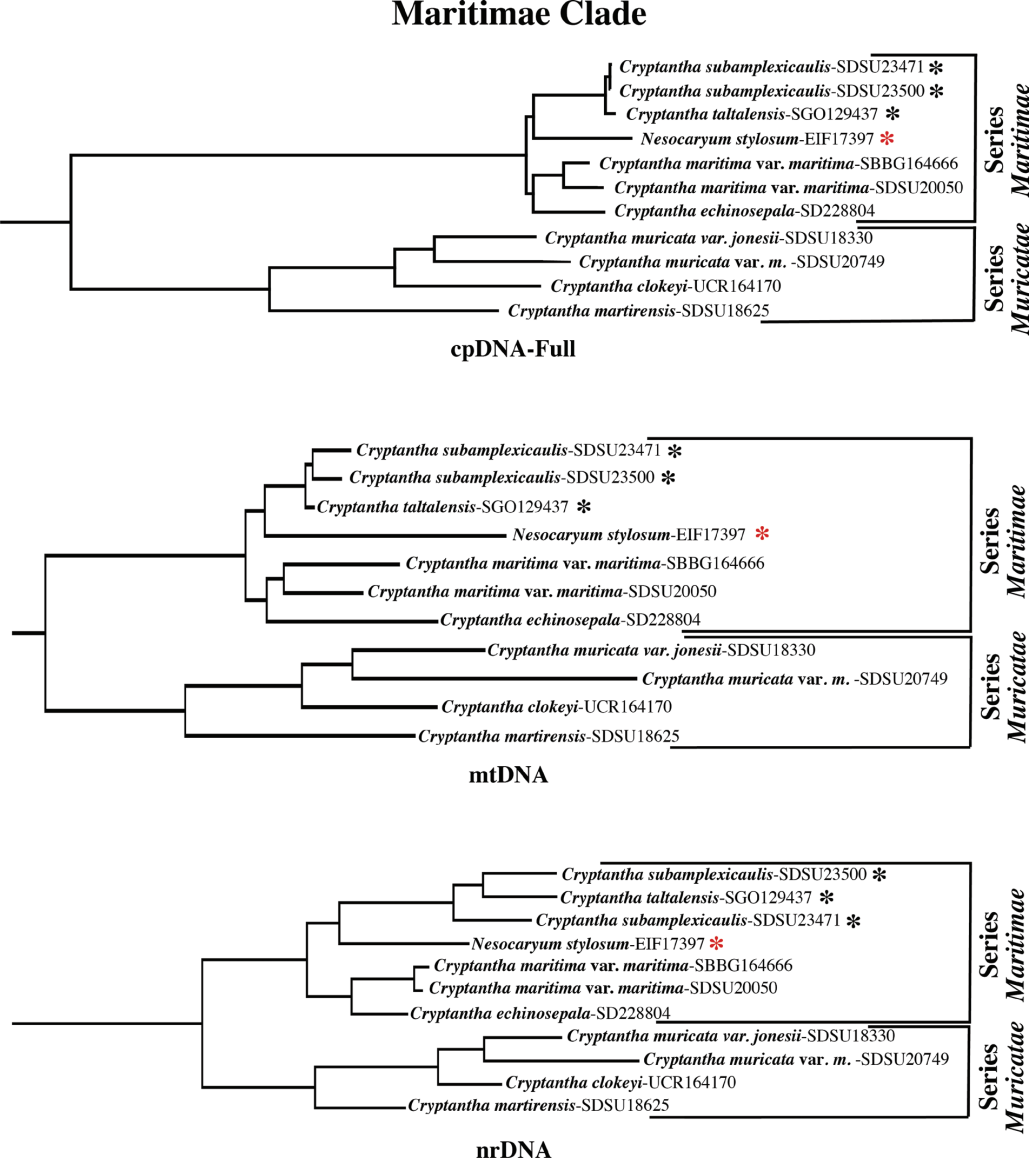
Phylogeny of the *Maritimae* clade in each of the three sequence analyses. Composition is identical across analyses, and topology is the same except for relationships of *C. taltalensis* and the two samples of *C. subamplexicaulis*, which differ in the nrDNA analysis. *Cryptantha* series *Maritimae* and series *Muricatae* are modified from Johnston (1925). *=South American taxa. All nodes have maximal bootstrap support. Abbreviations of DNA sequence types are as in Fig. 5.

In all three analyses, however, the *Maritimae* clade comprises the same nine species and 10 minimum-rank taxa, all of which exhibit the same topology except for *Cryptantha subamplexicaulis* and *C. taltalensis*, which show a slightly different topology in the nrDNA analysis (Fig. 6). The *Maritimae* clade consistently consists of two sister clades (Fig. 6), which correspond to the modified series *Maritimae* and series *Muricatae* of Johnston (1925). *Nesocaryum stylosum* is part of series *Maritimae* (Fig. 6), where it is sister to the South American species *Cryptantha subamplexicaulis* and *C. taltalensis*. These three species are sister to C. maritima var. maritima (two samples) and *C. echinosepala* J.F.Macbr. from North America. Series *Maritimae* is sister to a clade composed of *Cryptantha muricata* (Hook. & Arn.) A.Nelson & J.F.Macbr., *C. clokeyi* I.M.Johnst., and *C. martirensis* M.G.Simpson & Rebman, this clade corresponding to the series *Muricatae* (Fig. 6; see Discussion).

Given these sequence data and evidence from nutlet morphology, our results strongly support *Nesocaryum stylosum* as phylogenetically nested within series *Maritimae* of the *Maritimae* clade. In previous and current analyses, the *Maritimae* clade is either sister to *Cryptantha* s.s., representing the remainder of the genus, or more distantly related. We considered transferring the members of the *Maritimae* clade to another genus, but given the conflicting results, we preferred to defer this decision until additional sequence data—particularly nuclear DNA data—are available to better resolve the backbone relationships among clades of the Amsinckiinae. Thus, at this point, we consider it prudent to transfer *Nesocaryum stylosum* to the genus *Cryptantha*, given that: (1) the interrelationships of the *Maritimae* clade with other clades and genera vary depending on the sequence data analyzed; (2) the *Maritimae* clade is sister to *Cryptantha* s.s. in one analysis in the present study and in a separate analysis by Simpson et al. (2017a); (3) all other members of the *Maritimae* clade are currently classified in the genus *Cryptantha* (see Discussion); (4) the nutlet attachment scar of *N. stylosum* conforms to that of *Cryptantha* taxa; and (5) many members of the *Maritimae* clade still lack sequence data. We also note that members of the *Maritimae* clade lack an obvious morphological synapomorphy, other than a possibly elongate style, which is also present in some members of *Cryptantha* s.s.

Based on the total evidence and these considerations, we formally transfer *Nesocaryum stylosum* to *Cryptantha* as follows:

### ﻿Taxonomic treatment

#### ﻿ Cryptantha stylosa

Taxon classificationPlantaeBoraginalesBoraginaceae

(Phil.) Scherson, Nic.García & M.G.Simpson comb. nov.

19D45778-6973-524A-90AB-732214EFDED3

Heliotropium stylosum Phil., Bot. Zeitung (Berlin) 28: 500 (1870). Basionym.

##### Type.

Chile. Isla San Ambrosio, 1869, *Enrique Simpson s.n.* Holotype: barcode SGO000004198!=accession SGO054406!

## ﻿Discussion

As stated earlier, we confirm that this unusual plant is a member of *Cryptantha*, series *Maritimae* of the *Maritimae* clade of Simpson et al. (2017a) and Mabry and Simpson (2018) (Figs 5, 6). If future sequence data convincingly place the *Maritimae* clade apart from *Cryptantha* s.s., recognition of a separate genus for the clade may be warranted. For now, however, we retain the members of the *Maritimae* clade within the genus *Cryptantha*, treating this species as *C. stylosa*.

As noted above, the *Maritimae* clade in this study and in those of Simpson et al. (2017a) and Mabry and Simpson (2018) consists of two sister clades. One of these corresponds to the series *Muricatae* of Johnston (1925; Figs 1B, 6; Table 1). Johnston recognized only *Cryptantha muricata* (including its three varieties) in that series. We add four additional species to series *Muricatae*, resulting in a current composition of five species and seven minimum-rank taxa (Table 1; after Simpson et al. 2024). All of these taxa are morphologically similar in having ovoid fruiting calyces, ovate, papillate, and tuberculate to muricate nutlets, and a style extending to or beyond the nutlet apices (Simpson et al. 2024: Figs 4, 5).

**Table 1.** Taxa belonging to the *Maritimae* clade under the present classification, with eight species and 13 minimum-rank taxa in North America (NAm) and 12 species and minimum-rank taxa in South America (SAm), for a total of 19 species and 24 minimum-rank taxa (Cryptantha maritima var. pilosa occurs on both continents). *=Tentative placement in series after Simpson and Rebman (2021); **=tentative placement in series after Simpson et al. (2024). Taxa are grouped within series *Maritimae* and *Muricatae*, as circumscribed here. Indicated are taxa sequenced or analyzed in molecular phylogenetic studies of: 1 = Hasenstab-Lehman and Simpson (2012); 2 = Simpson et al. (2017a); 3 = Mabry and Simpson (2018); 4 = Böhnert, Borda Tamayo, Mabry, Schwarzer, Simpson, and Weigend (unpublished data); 5 = this paper.

**Series *Maritimae*-14 species, 17 minimum-rank taxa**		
*Cryptantha argentea* I.M.Johnst., Contr. Gray Herb. 78: 42 (1927)	SAm	
*Cryptantha chaetocalyx* (Philippi) I.M.Johnst., Contr. Gray Herb. 78: 43 (1927)	SAm	1,4
*Cryptantha echinosepala* J.F.Macbride, Contr. Gray Herb. 56: 57 (1918)	NAm	2,3,5
*Cryptantha filaginea* (Philippi) Reiche, Anales Univ. Chile 121: 829 (1907)	SAm	4
*Cryptantha filiformis* (Philippi) Reiche, Anales Univ. Chile 121: 829 (1907)	SAm	
*Cryptantha granulosa* (Ruiz & Pavon) I.M.Johnst., Contr. Gray Herb. 68: 54 (1923)	SAm	1
*Cryptantha limensis* (A.DC.) I.M.Johnst., Contr. Gray Herb. 70: 46 (1924)	SAm	
Cryptantha maritima (Greene) Greene var. cedrosensis (Greene) I.M.Johnst., Contr. Gray Herb. 74: 48 (1925)	NAm	
Cryptantha maritima (Greene) Greene var. maritima, Pittonia 1: 117 (1887)	NAm	1,2,3,4,5
Cryptantha maritima (Greene) Greene var. pilosa I.M.Johnst., Univ. Calif. Publ. Bot. 7: 445 (1922)	NAm & SAm	1
Cryptantha maritima (Greene) Greene var. vizcainensis Rebman & M.G.Simpson, Phytotaxa 509: 185–210 (2021)	NAm	
*Cryptantha pondii Greene, Pittonia 1: 291 (1889)	NAm	
*Cryptantha romanii* I.M.Johnst., Contr. Gray Herb. 78: 46 (1927)	SAm	
*Cryptantha stylosa* (Phil.) Scherson, Nic.García & M.G.Simpson, (this publication)	SAm	5
*Cryptantha subamplexicaulis* (Philippi) Reiche, Anales Univ. Chile 121: 826 (1907)	SAm	5
*Cryptantha taltalensis* I.M.Johnst., Contr. Gray Herb. 78: 45 (1927)	SAm	2,3,5
*Cryptantha varians* Brand, Repert. Spec. Nov. Regni Veg. 24: 57 (1927) [Note: Possibly = *C. granulosa*]	SAm	
**Series *Muricatae*-5 species, 7 minimum-rank taxa**		
*Cryptantha acrimuricata* J.André, L.M.Simpson & M.G.Simpson, Phytokeys 250: 193–213 (2024)	NAm	
*Cryptantha clokeyi* I.M.Johnst., J. Arnold Arbor. 20: 387 (1939)	NAm	2,3,5
**Cryptantha hooveri I.M.Johnst., J. Arnold Arbor. 18: 23 (1937)	NAm	
*Cryptantha martirensis* M.G.Simpson & Rebman, Madroño 60: 35–45 (2013)	NAm	2,3,5
Cryptantha muricata (Hooker & Arnott) A.Nelson & J.F.Macbride var. muricata, Botanical Gazette 61: 42 (1916)	NAm	2,3,5
Cryptantha muricata var. denticulata (Greene) I.M.Johnst., Contr. Gray Herb. 74: 71 (1925)	NAm	
Cryptantha muricata var. jonesii (A.Gray) I.M.Johnst., Pl. World 22: 114 (1919)	NAm	

The second clade within the *Maritimae* clade corresponds in part to the series *Maritimae* of Johnston (1925). Johnston recognized five species in this series: *Cryptantha dumetorum* (Greene ex A.Gray) Greene, *C. echinosepala*, *C. maritima*, *C. micromeres* (A.Gray) Greene, and *C. recurvata* Coville. Of these five species, the current study and those of Simpson et al. (2017a) and Mabry and Simpson (2018) support only two – *C. echinosepala* and *C. maritima* – as belonging to the series *Maritimae* (see Simpson and Rebman 2021); the other three species are more closely related to taxa or clades of *Cryptantha* s.s., which comprises the bulk of the genus (Simpson et al. 2017a; Mabry and Simpson 2018). Based on morphological similarities, Simpson and Rebman (2021) provisionally added one additional North American species – *Cryptantha pondii* Greene – and included all varieties of *C. maritima* (Table 1).

The molecular phylogenetic studies of Hasenstab-Lehman and Simpson (2012), Simpson et al. (2017a), Mabry and Simpson (2018), Böhnert, Borda Tamayo, Mabry, Schwarzer, Simpson, and Weigend (unpublished data), and the present study provide phylogenetic evidence for the inclusion of seven additional South American species in series *Maritimae* (see list in Table 1, including the variety of *C. maritima* occurring on both continents). With respect to *C. stylosa*, although Johnston (1927) recognized the similarity of its nutlets to those of *Cryptantha*, he chose to classify the species in a monotypic genus based on its unique habit, a shrub, and its distinctive calyx morphology, described as cylindrical, mostly synsepalous, with a ribbed tube and a linear, adnate floral bract. We interpret these features as autapomorphies of *Cryptantha stylosa*.

Ongoing morphological observations, based on nutlet sculpturing, nutlet attachment scar, and style length, allow us to provisionally add five additional South American species to the series *Maritimae*, bringing the preliminary total across the two continents to 14 species and 17 minimum-rank taxa (Table 1). A more detailed revision of the infrageneric classification of *Cryptantha* is currently underway [Simpson, Moroni, and Mabry (unpublished data)].

### ﻿Biogeographic history

**Figure 7.**
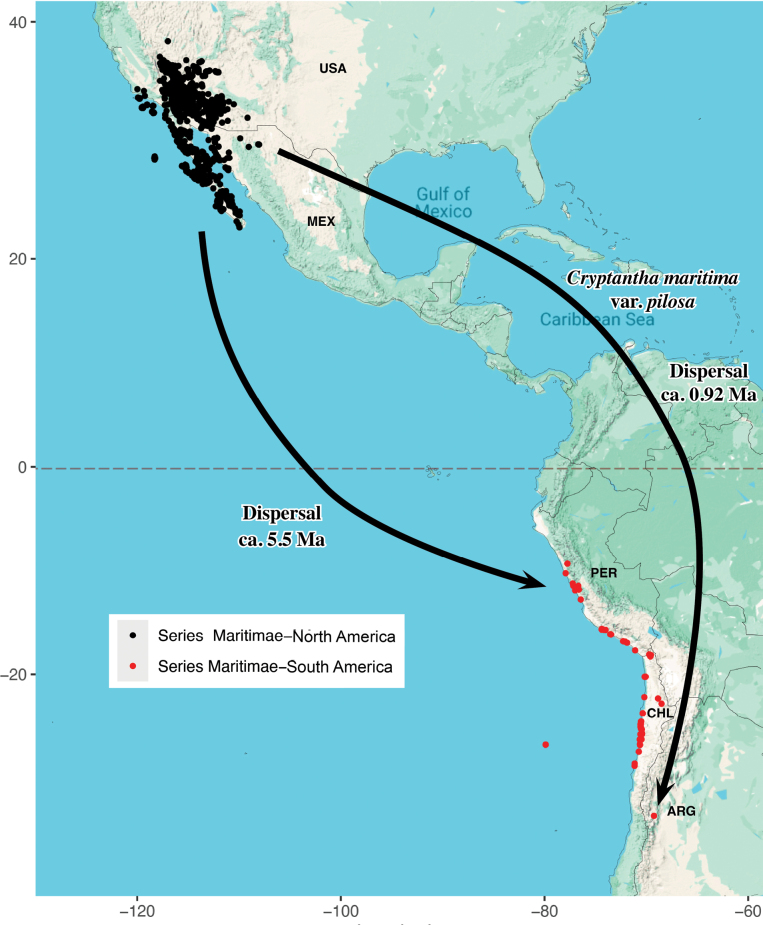
Distribution map of members of *Cryptantha* series *Maritimae* in North and South America, based on GBIF data [GBIF.org (2024a–p); see References], representing a total of 5,518 GBIF records; see Appendix 4 for listings by species and series. Two separate long-distance dispersal events and estimated times of dispersal are indicated (after Guilliams et al. 2017).

**Figure 8.**
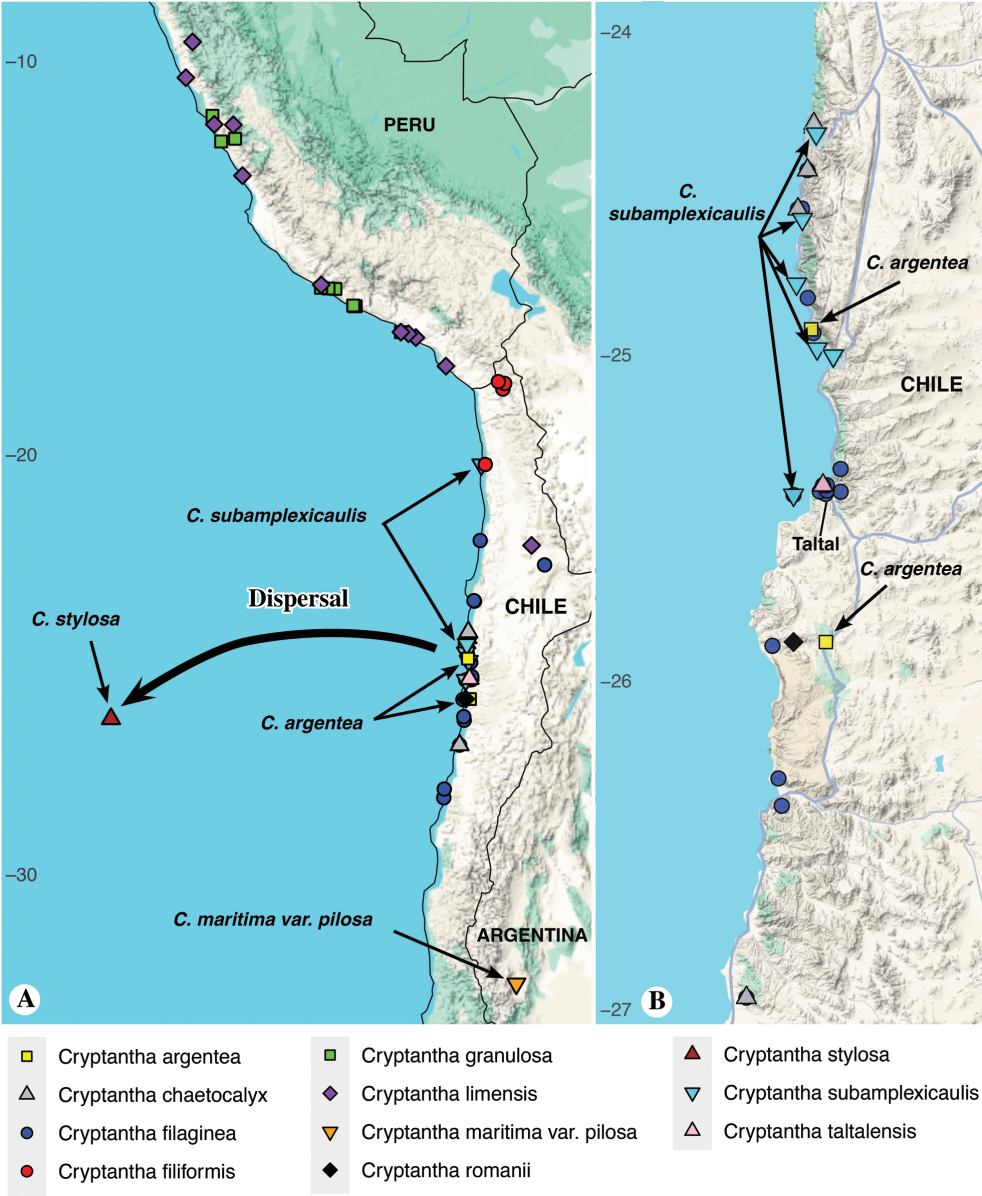
**A, B.** Close-ups of the distribution map showing localities of South American members of *Cryptantha* series *Maritimae*. **A.** Possible dispersal event from coastal Chile to Isla San Ambrosio; **B.** Larger-scale view of coastal Chile. Localities of *Cryptantha stylosa* and the putative close relatives *C. argentea* and *C. subamplexicaulis* are labeled.

The known distributions of members of series *Maritimae* are shown in Figs 7, 8 and exhibit a classic American amphitropic disjunct pattern (Guilliams et al. 2017; Simpson et al. 2017b). Although sequence data are lacking for many members of the series, the work of Guilliams et al. (2017) supports two past long-distance dispersal events within the series, both from western North America to western South America (Fig. 7). One dispersal event involved Cryptantha maritima var. pilosa I.M.Johnst., which is widespread in extreme southwestern North America, occurring in the Baja California Peninsula and Sonora, Mexico, as well as southeastern California, southern Nevada, and southern Arizona (see Simpson and Rebman 2021). The single known locality of this variety in northwestern Argentina (GBIF.org 2024i; see Figs 7, 8A) is estimated to have resulted from dispersal from North America approximately 0.92 million years ago (Guilliams et al. 2017).

A second, independent dispersal event within the series *Maritimae* is estimated to have occurred approximately 5.53 million years ago (Fig. 7). This estimate is approximate, as it is based on sequence data from only three species. Our working hypothesis is that this dispersal event led to a radiation in South America, culminating in the 11 species of the series, aside from C. maritima var. pilosa, that are currently recognized in that region. We further hypothesize that *C. stylosa* evolved following a single westward dispersal event across the Pacific Ocean from an ancestral mainland population (Fig. 8A; see below).

With the exception of the Andean C. maritima var. pilosa and the insular *Cryptantha stylosa*, the South American species of series *Maritimae* occur primarily in coastal regions from central Peru to central Chile, with a few populations recorded at higher elevations (Figs 7, 8). These coastal regions are relatively dry and receive little annual rainfall. However, they do receive precipitation via condensation of coastal fog, allowing coastal plant communities to persist (Moreira-Muñoz 2011).

### ﻿Taxonomic relationships of *C. stylosa* within series *Maritimae*

Given that eight of the 17 taxa of the *Maritimae* clade remain unsequenced, we currently lack a sufficient sample size to evaluate the phylogenetic relationships of *Cryptantha stylosa* to all other members of series *Maritimae*; however, support values within series *Maritimae* for the taxa that were sequenced are all above 90%. Based on morphology, we hypothesize that two other members of the series are its possible closest relatives: *C. argentea* I.M.Johnst. and *C. subamplexicaulis*. Both of these taxa, like *C. stylosa* and unlike other members of series *Maritimae*, are perennial. *Cryptantha subamplexicaulis* is described as “perennial or persistent annual with a firm branching root, erect and subsimple at first but later with several or many trailing branches from a loose fruticulose caudex and forming a loose prostrate mat 3–12 dm broad and 1–2 dm tall, hispid or hispid-villous and usually appressedly canescent” (Johnston 1927: 41). *Cryptantha argentea* is described as “perennial, prostrate, densely silvery-striated, with very numerous ramified branches from a prostrate, densely branched, shrubby stem rising into a cushion 2–5 cm high” and as “notable for its silvery, very dense strigose pubescence and particularly because of its very densely pulvinate [cushion-like] habit. The root is strong, branched, and indubitably perennial” (Johnston 1927: 42–43). We note that *C. argentea* was sequenced but was not included in any of the three analyses because of insufficient sequence data quality; samples were excluded from phylogenetic analyses if they had fewer than 60 loci recovered for the chloroplast gene dataset, 10 or fewer loci for the mitochondrial gene dataset, or fewer than 10 recovered regions in the full chloroplast genome assembly (see Appendix 3).

*Cryptantha stylosa*, as noted earlier, is also perennial but differs from both species in having an erect, woody, shrubby habit. *Cryptantha stylosa* resembles *C. subamplexicaulis* in having relatively large corollas (limb 6–7 mm wide in the former, 5–6 mm wide in the latter). However, it more closely resembles *C. argentea* in vestiture, with both species having a densely silvery-strigose stem and leaf indumentum. *Cryptantha stylosa* resembles both of these species, and all other South American members of series *Maritimae*, in having an elongate style that protrudes well beyond the nutlet apices; however, this character also occurs in some members of series *Muricatae*.

The woody, shrubby habit of *C. stylosa* represents a derived condition within the series *Maritimae* and is unique within the genus *Cryptantha*. Five other South American species of *Cryptantha* are perennial, but these are herbaceous and evolved independently, being members of distantly related clades of Johnston’s sections *Eucryptantha* and *Geocarya*, which are nested within *Cryptantha* s.s. (Johnston 1927; see Mabry and Simpson 2018). We interpret the unique habit of *C. stylosa* as an example of the evolution of “gigantism” following dispersal to an island, a pattern commonly involving evolutionary transitions to woody perennials (Carlquist 1966). Examples of this phenomenon include, within Boraginaceae, *Echium* in the Cape Verde Archipelago (Romeiras et al. 2011) and *Selkirkia* in the Juan Fernández Islands (Holstein et al. 2016), and, within Asteraceae, the Hawaiian silversword alliance – *Argyroxiphium*, *Dubautia*, and *Wilkesia* (Baldwin 2003).

## ﻿Conclusion

Based on the evidence presented here, *Nesocaryum stylosum* is placed in the genus *Cryptantha* as the new combination *C. stylosa* and is best classified within a revised and expanded series *Maritimae* of the *Maritimae* clade. Members of this series appear to have radiated in South America following two independent long-distance dispersal events: an early event resulting in diversification into 11 species of *Cryptantha* and a separate, relatively recent event involving C. maritima var. pilosa. The occurrence of *Cryptantha stylosa* on Isla San Ambrosio may represent yet another dispersal event from coastal Chile to the island, possibly involving ancestors related to the similarly perennial *C. argentea* and *C. subamplexicaulis*, although this hypothesis requires verification. Following dispersal to the island, the ancestor of *C. stylosa* underwent evolutionary changes in morphology, particularly the acquisition of a shrubby, woody habit that is unique within the genus and subtribe Amsinckiinae.

Although the current study provides a preliminary assessment of the composition and interrelationships of series *Maritimae*, additional work is needed to resolve this complex. Five South American species and three North American taxa remain unsequenced, and some South American samples were of limited quality. A more detailed molecular phylogenetic analysis of the group is required. Aside from *Cryptantha maritima*, the South American members of series *Maritimae* still require basic taxonomic study, including additional field collections and detailed analyses of morphological variation. Of the 14 species and 17 total taxa currently recognized in series *Maritimae*, only C. maritima var. pilosa has a published chromosome count (Las Peñas 2005); this count, 2n = 20, is aberrant within the genus. All other North American species have a chromosome number of 2n = 24, whereas non-hexaploid South American members of the genus have a chromosome number of 2n = 14 (see Amsinckiinae Working Group 2025). The reproductive biology and chemistry of members of the series remain unknown, with reproductive biology being of particular interest in understanding the adaptations of *C. stylosa* following its dispersal to an isolated island.

Future work incorporating expanded nuclear DNA sequence data will help determine whether the *Maritimae* clade is sister to *Cryptantha* s.s. and whether it should be recognized as a separate genus. Such studies will also provide the basis for a formal classification of the *Maritimae* clade and its infrageneric structure within the genus *Cryptantha*.

## Supplementary Material

XML Treatment for Cryptantha stylosa

## ﻿Additional information

### Use of AI

No use of AI was reported.

### Author ORCIDs

Michael G. Simpson https://orcid.org/0000-0002-6197-2132

Makenzie E. Mabry https://orcid.org/0000-0002-6139-9559

Paul D. Blischak https://orcid.org/0000-0001-9177-8958

Tim Böhnert https://orcid.org/0000-0003-1415-7896

C. Matt Guilliams https://orcid.org/0000-0003-3919-8432

Kristen E. Hasenstab-Lehman https://orcid.org/0000-0002-0277-5104

Federico Luebert https://orcid.org/0000-0003-2251-4056

Nicolás García https://orcid.org/0000-0001-9003-1510

Álvaro Castañeda https://orcid.org/0009-0005-2890-7012

Maximilian Weigend https://orcid.org/0000-0003-0813-6650

Rosa A. Scherson https://orcid.org/0000-0003-3207-6395

### Data availability

All of the data that support the findings of this study are available in the main text or Supplementary Information.

## ﻿Appendix 1

Protologue description of *Heliotropium stylosum* Phil. (1870: 500).

6. *Heliotropium stylosum* Ph. fruticosum, ramosissimum; foliis confertissimis, linearibus, basi sensim angustatis, apice subproductis, superne, supra medium sulcatis, pilos appressos strigosos gerentibus; floribus confertis; calyce brevi tubuloso, quinquefido, basi pilis albis densis obsito, laciniis glabriusculis; tubo corollae et calycem et limbum aequante; *stylo filiformi*, *ovarium bis aequante*.

Folia usque ad 7 lin. longa, 1½ lin. lata. Pedunculus communis 10 lin. longus, pilis albis strigosus; spicae breves, sed tantummodo primos flores explicatos gerebant, ideoque deterrninare non potui, utrum planta ad Heliophyta aut ad Heliotropia genuiua referenda sit. Squamae 5 in faucibus; antherae inclusae. Calyx 2 lin. longus; ovarium profunde quadrisulcatum; stigma parvum, peltatum. — Habitu simillimum *H. pyonophyllo* Ph. et *H. florido* DC.; prius differt tubo corollae calycem bis aequante, fere 4 lin. longo etc., *H. floridum* vero calyce omnino et aequaliter strigoso, corolla duplo majore, foliis multo latioribus etc.; praeterea stylus elongatus singularem characterem sistit.

**English translation (by Michael G. Simpson)**:

6. *Heliotropium stylosum* Ph. shrubby, much branched; leaves very dense, linear, lightly narrowed at the base, the apex slightly extended, furrowed above the middle, bearing appressed, strigose hairs; flowers clustered; calyx short tubular, pentamerous, base covered with dense white hairs, with slightly glabrous lobes; corolla tube and calyx and limb equal; style filiform, twice the ovary length.

Leaves up to 7 lin. [ca. 14 mm] long, 1½ lin. [ca. 3 mm] broad. Common peduncle 10 lin. [ca. 2 cm] long, with white strigose trichomes; spikes short, but they only bore the first flowers when they were opened, and therefore I could not determine whether the plant should be referred to as Heliophyta or as the genuine Heliotropia [*Heliotropium*?]. Scales five in the throat; anthers included. Calyx 2 lin. [ca. 4 mm] long; ovary deeply 4-divided; stigma small, peltate. — In habit very similar to *H.* [*Heliotropium*] *pycnophyllum* Phil. and *H.* [*Heliophytum*] *floridum* DC. [=*Heliotropium floridum* Clos]; from the first it differs by the tube of the corolla twice the length of the calyx, about 4 lin. [ca. 8 mm] long, etc., whereas *H. floridum* has a calyx completely and uniformly strigose, the corolla twice as large, the leaves much broader, etc.; moreover, the elongated style represents a unique character.

## Appendix 2

Protologue description of the genus *Nesocaryum* and the new combination *Nesocaryum stylosum* (Johnston 1927: 74–75).

10. ***Nesocaryum* gen. nov.**

Calyx cylindraceus costatus late et oblique affixis apice 5-dentatus fruticifer valde auctus latere abaxillare supra basem appendiculum (bracteam decurrentem) oblongum gerens. Corollae tubus breviter cylindricus calyci subaequalis intus infra medium cum 10 lamellis elongatis ornatus; lobis 5 ovatis imbricatis patentibus; fauce cum appendiculis 5 trapeziformibus instrusis clausa. Stamina 5 medio tubi affixa inclusa; filamentis brevissimis subulatis compressis; antheris oblongis obtusis quam filamenta multo longioribus. Stylus filiformis inclusis; stigmate capitato discoideo integerrimo. Ovulae 4. Semina recta, crassa; cotyledones integrae. Nuculae 4 erectae ovatae immarginatae tuberculatae latere interiore obtusae sulco medio-longitudinali basi furcato infra medium ad gynobasem subulatum affixae. —Fruticulus canescens insulae chilensis Sancti Ambrosiae dictae incola. Folia alterna oblanceolata breviter petiolata. Spicae geminatae. Corolla alba.— Nomen ex origine insulare a νησοξ, insula et καρυον, nux, derivatur.

**English translation (by Michael G. Simpson)**:

Calyx cylindrical, broadly, and obliquely costate, connate at the apex, 5-toothed, in fruit greatly enlarged laterally, bearing an oblong appendage (decurrent bract) above the base. Corolla tube shortly cylindrical, subequal inside, below the middle with 10 elongate lamellae; lobes five ovate, imbricate, horizontal; mouth closed with five inserted, trapeziform appendages. Stamen 5, affixed in the middle of the tube; filaments very short, compressed, subulate; anthers oblong, obtuse, much longer than filaments. Style filiform, included; stigma capitate, discoid, entire. Ovules 4. Seeds straight, thick; cotyledons entire. Nutlets 4, erect, ovate, unmargined, tuberculate, laterally obtuse ventrally, attached by a central longitudinal furrow to the base below the middle to the subulate gynobase. —A canescent shrub, native to the Chilean island of San Ambrosia. Leaves alternate, oblanceolate, shortly petiolate. Spikes twined. Corolla white.— The name is derived from its insular origin from νησοξ, island and καρυον, nut.

1. ***Nesocaryum stylosum*** (Ph.) comb. nov. Shrub (probably a small one), closely appressed canescent-strigose throughout; leaves oblanceolate, 2–4 cm long, 2–5 mm broad, veinless and rather firm, acute, strigose and finely pustulate, tapering off below into a short narrowly winged petiole; leaf-bases persistent and roughening the stem; spikes geminate, very densely flowered, ca. 1–1.5 cm long, rachis firm an tortuous, provided with long bracts decurrent on and coalescent with the base of the calyx; calyx firm, strigose, broadly obliquely and permanently attached to the rachis of the spike, on abaxial side above the base bearing a strict linear or narrowly spathulate appendage (the free end of the decurrent floral bract) reaching to the tip of the lobes; calyx at anthesis narrowly campanulate, ca. 3 mm long, lobed 1/3 to base; calyx at maturity decidedly accrescent, cylindrical, 6 mm long, lobed 1/5 to base, the tube ribbed; calyx lobes deltoid or at least triangular, unequal, usually green; corolla white, with a flat limb 6–7 mm broad; corolla tube about equaling calyx, ca. 3 mm. long, glabrous inside and out, ca. 1.2 mm. thick, slightly contracted towards the base, inside the lower 2/5 bearing five pairs of elongate protruding lamellae (a pair below each stamen-insertion) which are broadest (ca. 0.18 mm.) slightly thickened, and abruptly rounded off at the upper end and are gradually contracted in width towards the base of the tube; stamens borne about the middle of tube, ca. 0.7 mm below throat; filaments subulate, compressed, ca. 1/3–1/4 length of anthers; anthers oblong, obtuse, 0.8–1 mm. long; corolla-throat with five small (ca. 0.5 mm. long) emarginate glabrous trapeziform appendages of intruded tissue; corolla-lobes obovate, ca. 3 mm. long, rounded, spreading; nutlets (only slightly immature ones seen) 2–4, ovate-oblong, ca. 1.6 mm. long, ca. 0.6 mm. broad, minutely tuberculate, homomorphous, broadest ca. 0.5 mm. above base, gradually tapering above towards the acutish apex and rounded off below towards the truncate base, back convex, sides acute below and rounded towards apex, the groove open, narrow, abruptly dilated at forking to form a small deltoid areola; gynobase subulate, ca. 2/3–3/4 height of nutlets, 4-angled; style long, very much surpassing nutlets, ca. 1.5 mm. long.—*Heliotropium stylosum* Ph. Bot. Zeit. xxviii. 500 (1870) and Anal. Univ. Chile xlvii. 191 (1875); Hemsley, Bot. Challenger i. pt. 3, 100 (1884); Reiche in Eng. & Drude, Veg. Erde viii. [Grundz. Pflanzenverb. Chile] 269 (1907); Reiche, Anal. Univ. Chile cxxi. 832 (1908) and Fl. Chile v. 237 (1910).

CHILE: Isla San Ambrosio, August 1869, *Simpson* (MS [SGO], type; G [GH], photo.); Isla San Ambrosio, Sept. 1874, *Vidal* (MS; G. photo.).

A very peculiar insular monotype evidently derived from *Cryptantha* and characterized by its peculiar calyx and corolla-structures. The calyx is decidedly cylindrical, very broadly, obliquely, and firmly attached, and bears on the abaxial side above the base an elongate appendage. This appendage is evidently the floral bract that has become decurrent upon and fused with the lower part of the calyx. The corolla-tube below the middle on its inner surface is provided with five pairs of knife-like lamellae, which are apparently similar in origin to the minute scales frequently present at the base of the corolla-tube in *Cryptantha* and other genera. These unusual developments, the floral bracts decurrent on the calyx and the unusually large appendages of the corolla-tube, separate the proposed genus not only from *Cryptantha* but from practically all other genera of the subfamily.

Nesocaryum is a derivative of the section Krynitzkia of *Cryptantha* and probably from a member of the series *Barbigerae*. The fruit is indistinguishable from *Cryptantha* and quite like that of the large-flowered South American species of the series mentioned.

## ﻿Appendix 3

Added to those of Simpson et al. (2017a) in the present study. Listed are taxon, country, and state of origin of each sample, collector and collection number (in italics), herbarium accession (in parentheses), and NCBI Short Read Archive (SRA) run accession numbers. *=Specimen DNA quality did not meet the criteria for inclusion in analyses of all three DNA types; **=Specimen DNA quality did not meet the criteria for inclusion in cpDNA and nrDNA analyses.

***Cryptantha affinis*** (A.Gray) Greene, USA:California, *Simpson 4794*, (SDSU23665), SRR36494088; ***Cryptantha ambigua*** (A.Gray) Greene, USA:Oregon, *Halse 5442*, (RSA0444480), SRR36494087; ***Cryptantha aprica*** (Phil.) Reiche, Chile:Valparaíso, *Luebert 3744*, (SDSU23485), SRR36494076; ***Cryptantha arenophila*** Rebman & M.G.Simpson, Mexico:Baja California, *Rebman 33048*, (SDSU22932), SRR36494065; ****Cryptantha argentea*** I.M.Johnst., Chile:Antofagasta, *Johnston 5734*, (F625800), SRR36494054; ***Cryptantha aspera*** (Phil.) Grau, Chile:Coquimbo, *Luebert 3812B*, (SDSU23473), SRR36494043; ***Cryptantha barbigera*** (A.Gray) Greene var. ***barbigera***, USA:California, *Simpson 2793*, (SDSU17574), SRR36494032; ***Cryptantha barbigera*** (A.Gray) Greene var. ***barbigera***, USA:California, *Simpson 2789*, (SDSU19294), SRR36494021; ***Cryptantha barbigera*** (A.Gray) Greene var. ***barbigera***, USA:California, *Simpson 4864*, (SDSU25291), SRR36494010; ***Cryptantha barbigera*** (A.Gray) Greene var. ***furgusoniae*** J.F.Macbr., USA:California, *Simpson 5038*, (SDSU26284), SRR36493999; ***Cryptantha calycotricha*** I.M.Johnst., Chile:Valparaíso, *Luebert 3754B*, (SDSU23472), SRR36494086; ***Cryptantha catalinensis*** M.G.Simpson & Rebman, USA:California, *Hasenstab 4285A*, (SBBG), SRR36494085; ***Cryptantha catalinensis*** M.G.Simpson & Rebman, USA:California, *Hasenstab 4285A*, (SBBG), SRR36494084; ***Cryptantha catalinensis*** M.G.Simpson & Rebman, USA:California, *Guilliams 7289*, (SBBG251300), SRR36494083; ***Cryptantha clementina*** Rebman & M.G.Simpson, USA:California, *Hasenstab-Lehman 4718B*, (SBBG275035), SRR36494082; ****Cryptantha clementina*** Rebman & M.G.Simpson, USA:California, *Kaaret s.n.*, (SDSU23446), SRR36494081; ***Cryptantha clevelandii*** Greene var. ***clevelandii***, Mexico:Baja California, *Rebman 22927*, (SDSU20160), SRR36494080; ***Cryptantha clevelandii*** Greene var. ***clevelandii***, USA:California, *Simpson 4124*, (SDSU22659), SRR36494079; ***Cryptantha clevelandii*** Greene, USA:California, *Simpson 4730*, (SDSU23312), SRR36494078; ***Cryptantha clevelandii*** Greene, USA:California, *Hasenstab 4286*, (SBBG275032), SRR36494077; ****Cryptantha clevelandii*** Greene, USA:California, *Guilliams 6595*, (SBBG247970), SRR36494075; ***Cryptantha clevelandii*** Greene, USA:California, *Guilliams 5468*, (SBBG257325), SRR36494074; ***Cryptantha clevelandii*** Greene var. ***clevelandii***, USA:California, *Simpson 2923*, (SDSU19471), SRR36494073; ***Cryptantha clevelandii*** Greene, USA:California, *Simpson 4726*, (SDSU23308), SRR36494072; ***Cryptantha corollata*** (I.M.Johnst.) I.M.Johnst., USA:California, *Sanders 27455*, (RSA0094891), SRR36494071; ***Cryptantha crinita*** Greene, USA:California, *Ahart 10126*, (RSA0444551), SRR36494070; ***Cryptantha debilis*** (Phil.) Reiche, Chile:Tarapacá, *Luebert 3545B*, (SDSU23491), SRR36494069; ***Cryptantha decipiens*** (M.E.Jones) A.Heller, USA:California, *Simpson 2940*, (SBBG205104), SRR36494068; ***Cryptantha decipiens*** (M.E.Jones) A.Heller, USA:California, *Mabry 91*, (SDSU21234), SRR36494067; ***Cryptantha decipiens*** (M.E.Jones) A.Heller, USA:California, *Swinney 24686*, (SDSU25559), SRR36494066; ***Cryptantha dissita*** I.M.Johnst., USA:California, *Whipple 7626*, (SDSU23415), SRR36494064; ***Cryptantha echinella*** Greene, USA:California, *Bell 8409*, (RSA0097991), SRR36494063; ****Cryptantha flaccida*** (Douglas ex Lehmann) Greene, USA:California, *Carson 87*, (SBBG), SRR36494062; ***Cryptantha flaccida*** (Douglas ex Lehmann) Greene, USA:California, *Mason 214*, (SBBG254318), SRR36494061; ***Cryptantha foliosa*** (Greene) Greene, Mexico:Baja California, *Vanderplank 100505-10*, (RSA0072212), SRR36494060; ****Cryptantha foliosa*** (Greene) Greene, Mexico:Baja California, *Thorne 63103*, (RSA0074281), SRR36494059; ***Cryptantha ganderi*** I.M.Johnst., USA:California, *Hasenstab 40*, (SDSU20345), SRR36494058; ***Cryptantha ganderi*** I.M.Johnst., USA:California, *Simpson 4898A*, (SBBG), SRR36494057; *****Cryptantha ganderi*** I.M.Johnst., USA:California, *Simpson 4898B*, (SBBG), SRR36494056; ***Cryptantha gayi*** I.M.Johnst., Chile:Coquimbo, *Luebert 3778 B*, (SDSU23486), SRR36494055; ***Cryptantha globulifera*** (Clos) Reiche, Chile:Tarapacá, *Luebert 3641 B*, (SDSU23456), SRR36494053; ***Cryptantha glomerata*** Lehmann ex G. Don subsp. ***glomerata***, Chile:Valparaíso, *Luebert 3752 B*, (SDSU23480), SRR36494052; ***Cryptantha glomerata*** Lehmann ex G. Don subsp. ***quadrinuculata***, Chile:Coquimbo, *Luebert 3774 B*, (SDSU23481), SRR36494051; ***Cryptantha glomeriflora*** Greene, USA:California, *Simpson 5086*, (SDSU25344), SRR36494050; ***Cryptantha gracilis*** Osterhout, USA:California, *Andre 35004*, (RSA0141067), SRR36494049; ***Cryptantha grandiflora*** Rydb., USA:Idaho, *Kelley 1941*, (SDSU20620), SRR36494048; ***Cryptantha grandiflora*** Rydb., USA:Idaho, *Kelley 2057*, (SDSU20624), SRR36494047; ***Cryptantha hendersonii*** (A.Nelson) Piper ex J.C.Nelson, USA:California, *Janeway 12706*, (SBBG232175), SRR36494046; ***Cryptantha hendersonii*** (A.Nelson) Piper ex J.C.Nelson, USA:California, *Kelley 728*, (SDSU20590), SRR36494045; ***Cryptantha hendersonii*** (A.Nelson) Piper ex J.C.Nelson, USA:Oregon, *Kelley 854*, (SDSU20597), SRR36494044; ***Cryptantha hendersonii*** (A.Nelson) Piper ex J.C.Nelson, USA:Oregon, *Kelley 2194*, (SDSU20626), SRR36494042; ***Cryptantha hispidissima*** Greene, USA:California, *Hasenstab 29*, (SDSU18341), SRR36494041; ***Cryptantha intermedia*** (A.Gray) Greene var. ***intermedia***, USA:California, *Simpson 4191*, (SDSU22567), SRR36494040; ***Cryptantha intermedia*** (A.Gray) Greene var. ***intermedia***, USA:California, *Donovon 538*, (SDSU24688), SRR36494039; ***Cryptantha intermedia*** (A.Gray) Greene var. ***johnstonii*** J.F.Macbr., USA:California, *Simpson 4733*, (SDSU23313), SRR36494038; ***Cryptantha intermedia*** (A.Gray) Greene var. ***johnstonii*** J.F.Macbr., USA:California, *Simpson 4733*, (SDSU23313), SRR36494037; ***Cryptantha intermedia*** (A.Gray) Greene var. ***johnstonii*** J.F.Macbr., USA:California, *Swinney 18228*, (SDSU23887), SRR36494036; ***Cryptantha juniperensis*** R.B.Kelley & M.G.Simpson, USA:California, *House 1148*, (SDSU23622), SRR36494035; ***Cryptantha juniperensis*** R.B.Kelley & M.G.Simpson, USA:California, *Swinney 2491*, (SDSU25820), SRR36494034; ***Cryptantha kinkiensis*** Rebman & M.G.Simpson, USA:California, *Hasenstab 4791b*, (SBBG275031), SRR36494033; ***Cryptantha kinkiensis*** Rebman & M.G.Simpson, USA:California, *Hasenstab 4900*, (SBBG275034), SRR36494031; ****Cryptantha leiocarpa*** (Fischer & C.A.Meyer) Greene, USA:California, *Parikh 796*, (SBBG253811), SRR36494030; ***Cryptantha leiocarpa*** (Fischer & C.A.Meyer) Greene, USA:California, *Wilken 17927*, (SBBG253812), SRR36494029; ***Cryptantha macrocalyx*** (Phil.) Reiche, Chile:Atacama, *Simpson 3916*, (SDSU21626), SRR36494028; ***Cryptantha mariposae*** I.M.Johnst., USA:California, *Colwell 22-008*, (DAV241447), SRR36494026; ****Cryptantha mariposae*** I.M.Johnst., USA:California, *Colwell 22-012*, (DAV), SRR36494027; ***Cryptantha maritima*** (Greene) Greene var. ***maritima***, USA:California, *Hasenstab 1574*, (SBBG164666), SRR36494025; ****Cryptantha martirensis*** M.G.Simpson & Rebman, Mexico:Baja California, *Moran 31059*, (SBBG218546), SRR36494024; ***Cryptantha micromeres*** (A.Gray) Greene, USA:California, *Simpson 4737*, (SDSU23322), SRR36494023; ****Cryptantha microstachys*** (Greene ex A.Gray) Greene, USA:California, *Simpson 4925*, (SDSU26225), SRR36494022; ***Cryptantha milobakeri*** I.M.Johnst., USA:California, *Taylor 15786*, (SBBG161367), SRR36494020; ***Cryptantha mohavensis*** (Greene) Greene, USA:California, *Gardner 587*, (RSA0077399), SRR36494019; ***Cryptantha muricata*** (Hook. & Arn.) A.Nelson & J.F.Macbr. var. ***jonesii*** (A.Gray) I.M.Johnst., USA:California, *Hasenstab 18*, (SDSU18330), SRR36494018; **Cryptantha cf. nemaclada** Greene, USA:California, *Mabry 84*, (SDSU20776), SRR36494017; ***Cryptantha nevadensis*** A.Nelson & P.B.Kenn., USA:California, *Mabry 23*, (SDSU20361), SRR36494016; ***Cryptantha rattanii*** Greene, USA:California, *Simpson 3810*, (SDSU20735), SRR36494015; ***Cryptantha recurvata*** Coville, USA:California, *Andre 26277*, (SDSU20922), SRR36494014; ***Cryptantha simulans*** Greene, USA:California, *Bell 7208*, (RSA0040513), SRR36494013; ****Cryptantha* sp.**, USA:California, *Junak 6603*, (SBBG181122), SRR36494012; ***Cryptantha spithamaea*** I.M.Johnst., USA:California, *Colwell 22-006*, (DAV), SRR36494011; ***Cryptantha spithamaea*** I.M.Johnst., USA:California, *Colwell 21-026*, (SDSU23505), SRR36494009; ****Cryptantha spithamaea*** I.M.Johnst., USA:California, *Colwell 21-026*, (SDSU23505), SRR36494008; ***Cryptantha subamplexicaulis*** (Phil.) Reiche, Chile:Antofagasta, *Luebert 3973B*, (SDSU23471), SRR36494007; ***Cryptantha subamplexicaulis*** (Phil.) Reiche, Chile:Antofagasta, *Luebert 3931B*, (SDSU23500), SRR36494006; ***Cryptantha texana*** (A.DC.) Greene, USA:Texas, *Carr 25527*, (TEX00167487), SRR36494005; ***Cryptantha texana*** (A.DC.) Greene, USA:Texas, *Hansen 6609*, (TEX00460118), SRR36494004; ****Cryptantha torreyana*** (A.Gray) Greene var. ***torreyana***, USA:California, *Janeway 12746*, (SBBG233053), SRR36494003; ***Cryptantha watsonii*** (A.Gray) Greene, USA:California, *Honer 702*, (RSA0445570), SRR36494002; ***Cryptantha whippleae*** D.A.York & M.G.Simpson, USA:California, *Simpson 4760*, (SDSU23504), SRR36494001; ***Harpagonella palmeri*** A.Gray, USA:California, *Hasenstab-Lehman 4276*, (SBBG275033), SRR36494000; ***Johnstonella geohintonii*** (B.L.Turner) M.G.Simpson & R.B.Kelley, Mexico:NuevoLeon, *Hinton 28635*, (TEX00208442), SRR36493998; ***Johnstonella punensis*** M.G.Simpson & Muñoz-Schick, Chile:Antofagasta, *Teillier 3686*, (SGO139488), SRR36493997; ***Nesocaryum stylosum*** (Phil.) I.M.Johnst., Chile:Valparaíso, *Chamarro s.n.*, (EIF17397), SRR36493996; ****Pectocarya anomala*** I.M.Johnst., Chile:Tarapacá, *Luebert 3597 B*, (SDSU23578), SRR36493995;

## Appendix 4

Total numbers of records obtained from GBIF (see GBIF.org 2024a–p) for mapping taxa, for a grand total of 5,518 records.

**Series *Maritimae* -14 species, 17 minimum-rank taxa**

*Cryptantha argentea* 2

*Cryptantha chaetocalyx* 6

*Cryptantha echinosepala* 124

*Cryptantha filaginea* 32

*Cryptantha filiformis* 4

*Cryptantha granulosa* 15

*Cryptantha limensis* 11

*Cryptantha maritima* (all 4 varieties) 2,456

*Cryptantha pondii* 16

*Cryptantha romanii* 1

*Cryptantha subamplexicaulis* 6

*Cryptantha taltalensis* 1

*Nesocaryum stylosum* 4

Total: **2,678**

**Series *Muricatae* -5 species, 7 minimum-rank taxa**

*Cryptantha clokeyi* 69

*Cryptantha martirensis* 72

*Cryptantha muricata* (all 3 varieties) 2,699

Total: **2,840**

## ﻿Appendix 5

iNaturalist (https://www.inaturalist.org) observations of *Nesocaryum stylosum* by Lukas Mekis (lukasmekis), lukasmekis, some rights reserved (CC BY-NC), including the dates and georeferenced coordinates of these observations.

https://www.inaturalist.org/observations/38141792, lukasmekis, 9-Sep-2018, -26.34736, -79.88904

https://www.inaturalist.org/observations/23380789, lukasmekis, 10-Sep-2018, -26.34738, -79.89309

https://www.inaturalist.org/observations/103881728, lukasmekis, 17-Dec-2021, -26.34769, -79.89408

https://www.inaturalist.org/observations/112149284, lukasmekis, 31-Mar-2022, -26.34713, -79.8900

## Supplementary materials

Supplementary material 1 Cladogram of the whole chloroplast (cpDNA) dataset for all taxa analyzed, with bootstrap values shown at nodes https://doi.org/10.3897/phytokeys.269.176263.suppl1 Data type pdf https://binary.pensoft.net/file/1510781This dataset is made available under the Open Database License (http://opendatacommons.org/licenses/odbl/1.0/). The Open Database License (ODbL) is a license agreement intended to allow users to freely share, modify, and use this Dataset while maintaining this same freedom for others, provided that the original source and author(s) are credited.Michael G. Simpson, Makenzie E. Mabry, Paul D. Blischak, Tim Böhnert, C. Matt Guilliams, Kristen E. Hasenstab-Lehman, Federico Luebert, Nicolás García, Álvaro Castañeda, Maximilian Weigend, Rosa A. Scherson

Supplementary material 2 Cladogram of the mitochondrial (mtDNA) dataset for all taxa analyzed, with bootstrap values shown at nodes https://doi.org/10.3897/phytokeys.269.176263.suppl2 Data type pdf https://binary.pensoft.net/file/1510782This dataset is made available under the Open Database License (http://opendatacommons.org/licenses/odbl/1.0/). The Open Database License (ODbL) is a license agreement intended to allow users to freely share, modify, and use this Dataset while maintaining this same freedom for others, provided that the original source and author(s) are credited.Michael G. Simpson, Makenzie E. Mabry, Paul D. Blischak, Tim Böhnert, C. Matt Guilliams, Kristen E. Hasenstab-Lehman, Federico Luebert, Nicolás García, Álvaro Castañeda, Maximilian Weigend, Rosa A. Scherson

Supplementary material 3 Cladogram of the nuclear ribosomal (nrDNA) dataset for all taxa analyzed, with bootstrap values shown at nodes https://doi.org/10.3897/phytokeys.269.176263.suppl3 Data type pdf https://binary.pensoft.net/file/1510783This dataset is made available under the Open Database License (http://opendatacommons.org/licenses/odbl/1.0/). The Open Database License (ODbL) is a license agreement intended to allow users to freely share, modify, and use this Dataset while maintaining this same freedom for others, provided that the original source and author(s) are credited.Michael G. Simpson, Makenzie E. Mabry, Paul D. Blischak, Tim Böhnert, C. Matt Guilliams, Kristen E. Hasenstab-Lehman, Federico Luebert, Nicolás García, Álvaro Castañeda, Maximilian Weigend, Rosa A. Scherson
