# Supplementary figures and images for: ﻿Resolving the taxonomic enigma of Nesocaryum stylosum (Boraginaceae): phylogenetic evidence for its reclassification as Cryptantha stylosa
Source: PhytoKeys. 2026 Jan 13;269:141–69. doi: 10.3897/phytokeys.269.176263 (PMC12820567; doi:10.3897/phytokeys.269.176263)

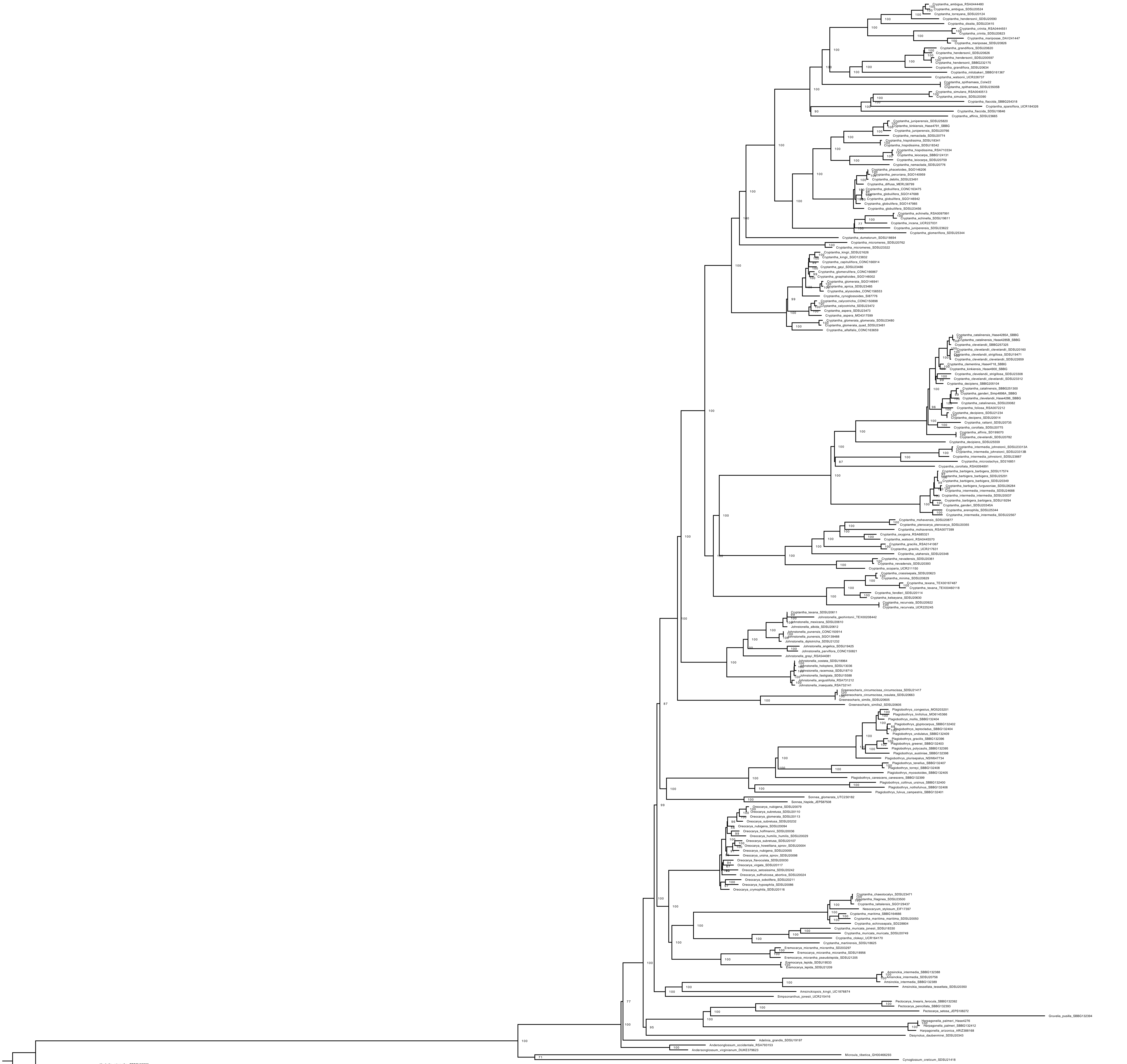

Supplement: Supplementary material 1 — Cladogram of the whole chloroplast (cpDNA) dataset for all taxa analyzed, with bootstrap values shown at nodes [file phytokeys-269-141_article-176263__-s001.pdf]

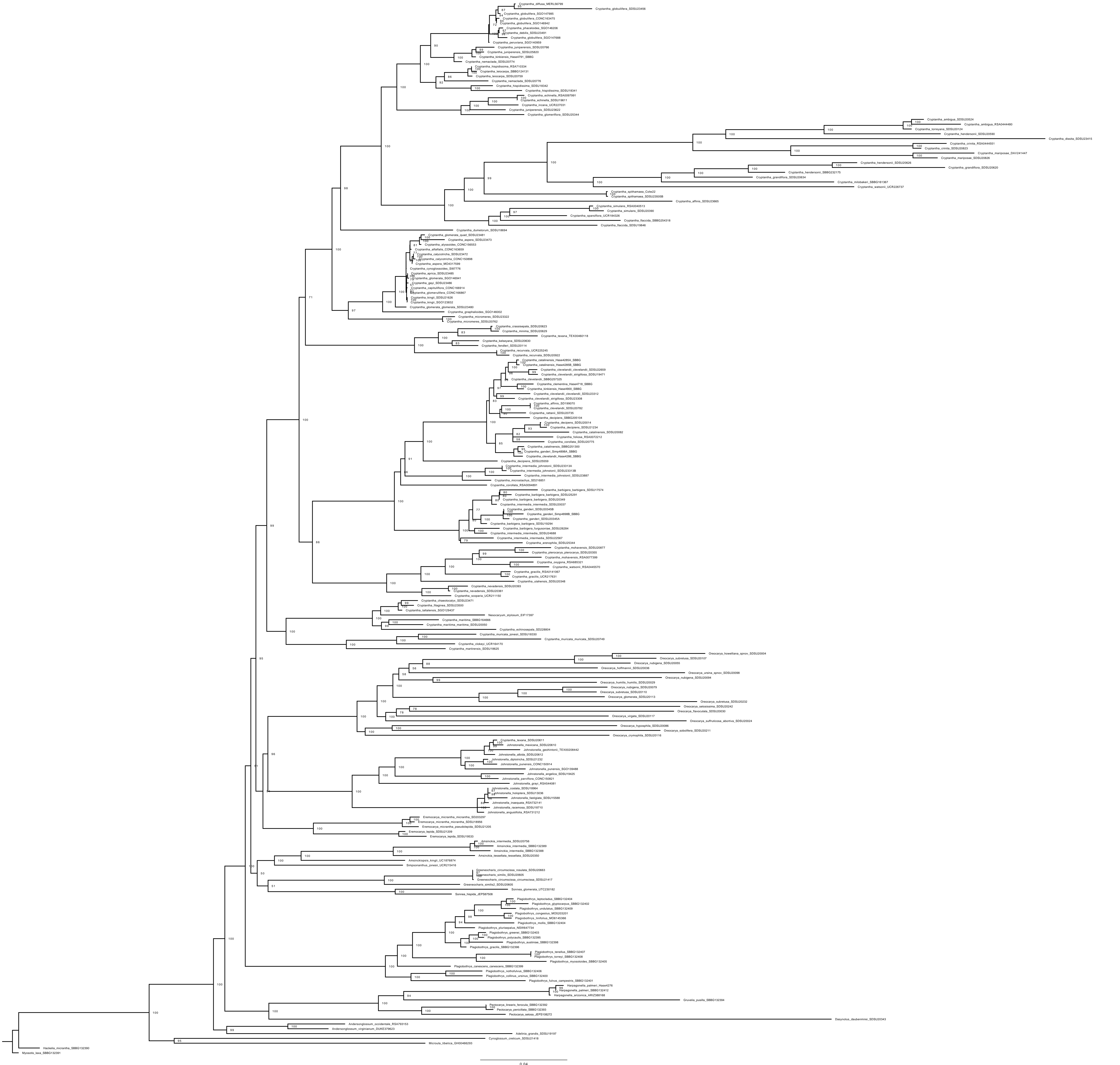

Supplement: Supplementary material 2 — Cladogram of the mitochondrial (mtDNA) dataset for all taxa analyzed, with bootstrap values shown at nodes [file phytokeys-269-141_article-176263__-s002.pdf]

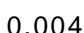

Supplement: Supplementary material 3 — Cladogram of the nuclear ribosomal (nrDNA) dataset for all taxa analyzed, with bootstrap values shown at nodes [file phytokeys-269-141_article-176263__-s003.pdf]
